# Exploring DNA Methylation Diversity in the Honey Bee Brain by Ultra-Deep Amplicon Sequencing

**DOI:** 10.3390/epigenomes4020010

**Published:** 2020-06-25

**Authors:** Robert Kucharski, Ryszard Maleszka

**Affiliations:** 1 Research School of Biology, Australian National University, Canberra, ACT 2601, Australia; robert.kucharski@anu.edu.au; 2 Faculty of Science and Technology, University of Canberra, Canberra, ACT 2617, Australia

**Keywords:** DNA methylation, epialleles, brain epigenome, cellular diversity, social insect, gene expression, nadrin, dynactin

## Abstract

Understanding methylation dynamics in organs or tissues containing many different cell types is a challenging task that cannot be efficiently addressed by the low-depth bisulphite sequencing of DNA extracted from such sources. Here we explored the feasibility of ultra-deep bisulphite sequencing of long amplicons to reveal the brain methylation patterns in three selected honey bee genes analysed across five distinct conditions on the Illumina MiSeq platform. By combing 15 libraries in one run we achieved a very high sequencing depth of 240,000–340,000 reads per amplicon, suggesting that most of the cell types in the honey bee brain, containing approximately 1 million neurons, are represented in this dataset. We found a small number of gene-specific patterns for each condition in individuals of different ages and performing distinct tasks with 80–90% of those were represented by no more than a dozen patterns. One possibility is that such a small number of frequent patterns is the result of differentially methylated epialleles, whereas the rare and less frequent patterns reflect activity-dependent modifications. The condition-specific methylation differences within each gene appear to be position-dependent with some CpGs showing significant changes and others remaining stable in a methylated or non-methylated state. Interestingly, no significant loss of methylation was detected in very old individuals. Our findings imply that these diverse patterns represent a special challenge in the analyses of DNA methylation in complex tissues and organs that cannot be investigated by low-depth genome-wide bisulphite sequencing. We conclude that ultra-deep sequencing of gene-specific amplicons combined with genotyping of differentially methylated epialleles is an effective way to facilitate more advanced neuro-epigenomic studies in honey bees and other insects.

## 1. Introduction

Tissues with a high level of cellular complexity, such as the brain, represent a special challenge in epigenomic analyses [1,2]. Although many brain cell types can be easily defined on the basis of their morphology, connectivity and specific locations or transmitter repertoires, we are still largely ignorant of how many functionally distinct cells may exist even in a relatively simple invertebrate brain [3]. In *Drosophila*, an old assessment that the intrinsic neurons (Kenyon cells) of the brain centres, known as mushroom bodies, are functionally identical isomorphic arrays, was radically reshaped following the development of the enhancer trap-based methodology [4]. It has been shown that these cells form parallel sub-compartments, exhibiting discrete patterns of gene expression [4]. Similarly, distinct patterns of spatial expression of cell type-specific genes have been found in the main compartments and small regional subdivisions of the mouse brain [5]. At the epigenomic level, the potential of differentially methylated genomic regions to define functionally distinct cell types was investigated in the human brain [6]. These findings suggest that subsets of morphologically similar brain cells could have diverse “molecular identities”, established either during development or as a result of activity-dependent changes, or both, and may specify the functionally distinct cell types of the neurons and glia.

Although much progress has been made in unravelling the basic aspects of epigenomic modifications in mammals, the sheer complexity of mammalian brains combined with their heavily methylated genomes represents a major experimental impediment in these studies, in particular at the level of functional cell-type resolution [7,8,9,10]. This is an area in which excellent progress can be made using smaller brains of sophisticated insects, such as the social honey bee, *Apis mellifera* [3,11,12].

The discovery of a DNA methylation system in the honey bee [12,13] has created an excellent opportunity to study DNA methylation dynamics in a sophisticated organism with a compact, sparsely methylated genome and a relatively small, yet sufficiently complex brain that controls an advanced behavioural repertoire, including navigational skills, long term memory and the only known non-primate symbolic “language” [14]. The seminal mapping of the whole adult brain and larval head methylomes in honey bee queens and workers provides the initial impetus for the emerging field of comparative neuro-epigenomics [15,16]. However, it became clear that in this polyandrous organism a high proportion of differential methylation is driven by obligatory epialleles, whose impact on genome regulation is poorly understood [8,17,18].

We hypothesized that functionally distinct cell types in the insect brain could be identified on the basis of the methylation patterns of illustrator genes generated by sequencing of long amplicons at high depth. In contrast to the low-depth (5–20× coverage) genome-wide methylation profiles that are assembled from short 75–100 base pairs (bp) reads, representing the combined methylomes of many different cell types, Illumina platforms such as MiSeq or NovaSeq allow for analysing sequence lengths of ~600bp and at a depth of many thousand reads per amplicon [19]. Assuming predominantly symmetrical CpG methylation in the honey bee [20], and each read representing a single-strand DNA methylation event in one chromosome per nucleus, the resolving power of this approach has the potential to visualize all condition-specific patterns that may be associated with epigenetically distinct cell types. Furthermore, many conditions can be sequenced in parallel in one run.

Here, we have asked how many diverse methylation patterns can be detected in the honey bee brain during various stages of adult maturation and in individuals preforming different tasks. Three conserved genes expressed in the brain with a relatively high number of confirmed methylated CpGs were selected as illustrators of methylation changes (Table 1). The five chosen conditions represent important situations of great interest for studies on the behaviour and adult maturation in honey bees that are either shown or predicted to be epigenetically controlled [8,15,16,21] (Table 2): (1) 7-day-old bees reared in a dark incubator with very little life experience and no colony-level experience; (2) 14-day-old bees getting ready for a major life transition, namely nurse-to-forager. These bees undertook orientation flights and their brains have the initial activity-dependent imprints driven by light and spatial learning; (3) and (4) two groups of 93-day-old bees, representing two distinct functional categories, namely nurses and foragers. This is a most interesting group that allows for exploring differences driven by task rather than age; and (5) very old bees that were selected to examine the potential effect of age on methylation. We show that distinct patterns of methylated CpGs in the honey bee brain are easily detectable in various situations by employing ultra-deep amplicon sequencing. We argue that the significance of DNA methylation with respect to behaviour and adult maturation can be effectively studied by this approach.

## 2. Results

Both the quality and quantity of reads generated by Illumina MiSeq in our experiments have consistently been excellent, yielding 240,000–340,000 reads per amplicon. Although like in other species, the number of cell types in the honey bee brain is not known, and such coverage and the length of amplicons ensures that most if not all methylation patterns potentially associated with a given gene/condition can be visualised in a population of around 1 million neurons [22].

**Figure 1:**
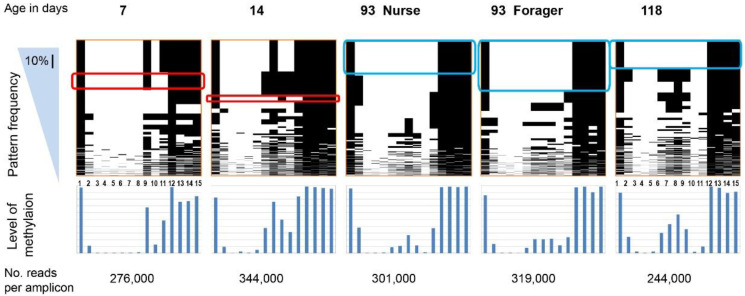
All methylation patterns for 15 CpG sites in the *nadrin* amplicon revealed by Illumina MiSeq; “Frequency” denotes the pattern sorting direction (i.e., most frequent patterns at the top). The scale bar in the blue triangle corresponds to 10% of all patterns. The lower panel shows the combined methylation level for each CpG. The rounded red and blue rectangles indicate selected identical patterns between two or three situations. The age of the bees used is shown above the top panel (see Table 2 for a full description of the biological material). For details on the *nadrin* gene structure and amplicon localisation, see Figure S1 and Table 1. The number of reads per amplicon is indicated at the bottom.

The three genes selected for this project, *dynactin*, *nadrin* and *pkcbp1* (Table 1 and Figures S1–S3), have previously been shown to be methylated by the whole genome bisulphite sequencing [15,19]. All three genes have a relatively high number of methylated CpGs (Table 1 and Figure 1, Figures S4 and S5) and produce alternatively spliced transcripts. Some correlative evidence from the honey bee transcriptome and methylomes suggest that methylated CpGs may play a role in alternative splicing. In the case of pkcbp1, two methylated CpGs have been implicated in generating two splice variants encoding distinct protein isoforms (Figure S3). All three genes encode proteins with known roles in various organisms and given their high level of conservation are predicted to have similar roles in the honey bee brain, namely cytoskeletal rearrangements, cell differentiation and cellular signalling [19,21].

**Table 1:** Description of the amplicons. The table shows the NCBI accession numbers, exons covered by nested PCR products and nested product lengths created using the primer sets described previously [23].

Gene	NCBI IDs	Analysed Exons	Amplicon Length (bp) **	mCpGs/ Amplicon	Comments
*Dynactin* * dctn4 subunit 4	XP_006560650	4, 5, 6	551	10	Differentially spliced, methylated in queen and worker adult brain and in larval head
*Nadrin*, rho GTPase-activating protein	XP_006572269	10	405	15	Differentially spliced, methylated in queen and worker brain
*PKCbp1*, protein kinase C-binding protein	XP_016769618	5, 6	401	10	Methylated gene, different isoforms expressed in adult brains and larval heads

* The BeeBase annotation of Dynactin shows a gene model with 10 exons, whereas the NCBI shows a gene model with 9 exons. The extra exon in BeeBase is located between NCBI exons 7 and 8. Our RNA seq data support the NCBI model (see Figure S2 for dctn4 gene model and References [16] and [24] for accession numbers). ** Not including adaptors and indices.

**Figure 2:**
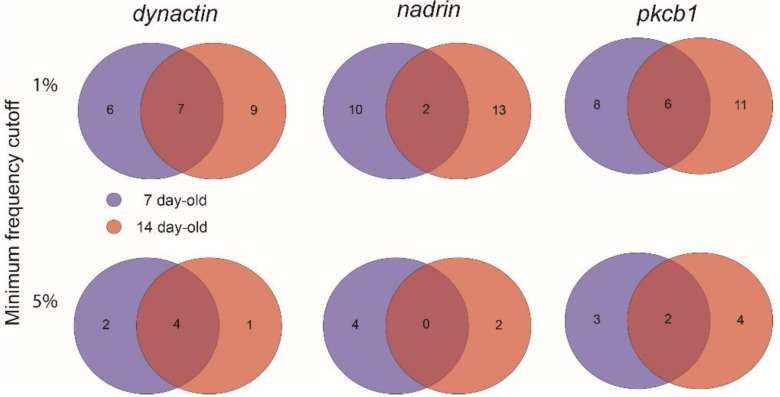
A 2-way Venn diagram showing the number of unique and overlapping patterns for *dynactin*, *nadrin* and *pkcb1* in 7-day-old and 14-day-old bees (1% and 5% minimum frequency cut-off).

**Figure 3:**
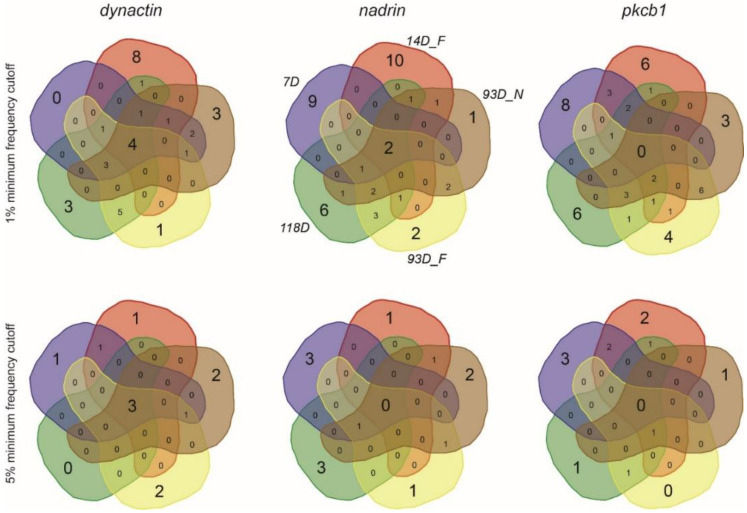
Symmetrical Venn diagrams showing the number of unique and overlapping patterns for *dynactin*, *nadrin* and *pkcb1* from all the analysed conditions (1% and 5% minimum frequency cut-off).

**Figure 4:**
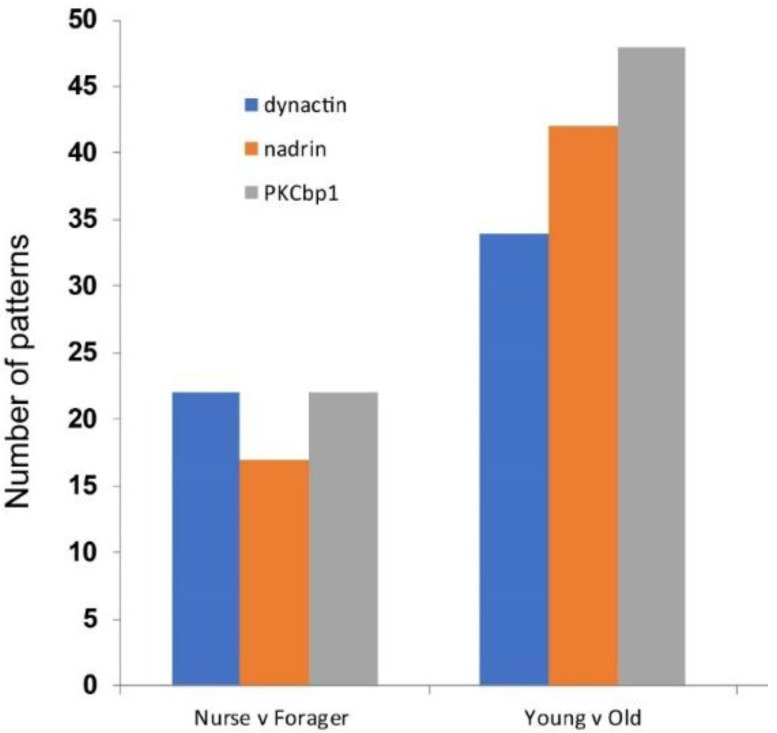
Number of cumulative patterns for *dynactin*, *nadrin* and *pkcb1* with a minimum 1% frequency, 2-way comparisons: nurses versus foragers (all 93-day-old bees) and young bees (combined 7- and 14-day-old bees) versus old bees (combined 93-day-old nurses and foragers and 118-day-old bees).

Figure 1 shows the patterns of *nadrin* methylation in the honey bee brain across all five analysed conditions, including bees of different ages and experience. Interestingly, we have found a tendency of certain CpG sites to show high methylation dynamics (e.g., CpG #11) with other sites remaining almost unchanged as unmethylated (e.g., CpG #3) or methylated (e.g., CpG #12). In the case of *nadrin*, the middle part of the analysed gene fragment shows higher methylation dynamics than the flanking regions, which appear to change only moderately (Figure 1). The methylation of the other two analysed genes, *dynactin* and *pkcbp1*, follows a similar arrangement with the majority of the detected CpGs showing little dynamics. While the bulk of the condition-specific patterns is represented by only a few configurations of methylated CpGs, there are additional less-frequent patterns detectable in each situation. At this stage it is difficult to determine if these rare patterns are genuine or if they represent technical noise, resulting, for example, from the nature of the sequencing technology. Alternatively, these less frequent patterns could represent changes in methylation driven by behavioural activities or external stimuli. Such activity-dependent modification take place only in restricted areas of the brain and therefore are intermittent [8,14]. Applying different pattern frequency cut-offs reduces the number of both unique patterns and overlapping patterns between different situations. However, the overall conclusions remain unchanged regardless of the applied cut-offs. One example of a 2-way comparison of *nadrin* methylation patterns in 7-day- and 14-day-old bees is shown in Figure 2. Perhaps not surprisingly, the most similar patterns were found in individuals of the same age (93 days) but performing distinct tasks (nurses vs. foragers). The Venn diagrams in Figure 3 show a compilation of all the patterns in the three analysed genes across all five conditions. The number of overlapping patterns is minimal, even with the 1% frequency cut-off. Our data also confirm that there are more methylation changes associated with age than with tasks. The number of age-related patterns for young and old doubles the number of patterns found in nurses and foragers of the same age (Figure 4). With regard to age, we find only a moderate loss of the overall methylation in 93-day-old bees versus 7- and 14-day-old bees, with no further decrease in the oldest individuals (118-day-old bees).

## 3. Discussion

### 3.1. What Is the Meaning of the Novel Methylation Patterns Uncovered by this Study?

There are several important conclusions emerging from this study. First, the number of condition-specific methylation patterns is low, suggesting that they may correlate with the epigenetic polymorphism (epialleles) in individuals analysed in each group. Indeed, the combined contribution of maternal/paternal patterns in the pooled sample of five bees used for each situation is expected to roughly yield the observed frequency of the main patterns. The relevance of the genetic polymorphism to methylomics in this polyandrous organism was highlighted in a couple of recent studies [18,25]. These studies suggest that the obligatory epialleles arising from sequence variations are most likely the main source of differential methylation between various situations previously found in the honey bee [15,16]. In this context, the mapping of any potential de novo methylation marks driven by brain activities poses a greater challenge. It is conceivable that in our dataset such a limited de novo methylation is represented by the infrequent patterns. This notion is supported by our recent finding that the full extent of CpG methylation in a gene encoding AmDAT (dopamine transporter) could only be revealed by ultra-deep sequencing [26]. Given the restricted expression of AmDAT in just four clusters of neurons in the honey bee brain [27], this result is not surprising, and in addition, it reinforces the idea that at least in some cases DNA methylation is positively correlated with transcription [25]. Since the overall number of patterns in the honey bee brain is manageable, the contribution of epialleles and de novo methylation to the honey bee brain methylomes can be experimentally resolved by deep amplicon sequencing combined with genotyping. Secondly, there is very little overlap between the patterns seen in the different conditions with both age and function correlating with the different patterns. Thirdly, certain CpG dinucleotides are more prone to methylation changes than others. The extent to which the observed methylation patterns are relevant to brain function cannot be clearly established without a full understanding of the effects of individual CpGs on gene expression. One possibility is that such variable differentially methylated regions represent hotspots for interactions with chromatin [28] or microRNAs [24] and may be important for transcript quality and/or quantity control [17,25,29,30,31]. Like in many other honey bee genes, some of the methylated CpGs analysed in this study are localised in the region where alternatively spliced variants are generated (Figure S3). Alternatively, changes in the methylation status of the CpGs in a given region may affect the level of transcription in accord with the idea that methylation alone or in combination with chromatin modifications can modulate gene expression [17,28]. In the mouse, non-promoter DNA methylation may be used for maintaining the active chromatin states of genes critical for development [32], and in *Arabidopsis*, DNA methylation can act to fine-tune gene expression by balancing the repressive and activating transcriptional effects [33]. An alternative explanation is that in a germ line this could be a way of “registering” activity and transferring this mark to the next generation [8,18]. By employing the strategy outlined in the present study, it should be possible to understand the extent of the fluidity and possibly the stochasticity as well as hereditability of the DNA methylation patterns in the honey bee, and the role they may play in behaviour and development.

### 3.2. Experimental Inferences

The massively parallel amplicon sequencing allows for analysing methylation patterns in genes of interest across several conditions, even if the methylation levels in certain cell types may be very low. Another advantage over low-coverage genome-wide bisulphite sequencing is that a given pattern is not generated from a small number of shorts reads, and thus different cells indicate only the cumulative levels of methylation at a given CpG rather than the individual cell-type patterns. In contrast, specific methylation of many CpGs in a ~600 bp region can be revealed with a depth of several thousand reads per one amplicon for thousands of cells. Similar sequencing depths of long DNA fragments can also be achieved on newer NGS platforms, such as NovaSeq, but such options are still more expensive than MiSeq.

Although the general levels of methylation in individual CpGs are similar to those found in previous genome-wide mapping in this species [15,16], the actual patterns are novel because such patterns cannot be inferred from short sequencing reads obtained at a much lower depth and then mapped onto a genome assembly. Previously available methylation maps for a given genomic region represented combined patterns of many cells. In some studies, the sequencing depth was only 5–10× per read, with occasional efforts reaching 10–20x coverage [19]. While such methylomes are useful in uncovering the methylated regions, they offer little information about condition-specific methylation dynamics across diverse cell types. In the present study, the coverage of at least 240,000 reads per amplicon reveals a number of previously undetectable patterns that might delineate potential hotspots of methylation dynamics. Although most of these differentially methylated patters are likely to be generated by epiallelic differences, the less frequent patterns may represent de novo methylation/demethylation. Regardless of the means by which these diverse methylation patterns are produced, their impact on gene expression is predicted to be important in various contexts. Previous findings in plants and mammals suggest that DNA methylation variation is influenced by genetic and epigenetic changes that are often stably inherited and can influence the expression of nearby genes [29,31].

We show that a massively parallel sequencing of relatively long amplicons with Illumina MiSeq technology can be used to reveal the methylation patterns of specific genes at a previously inconceivable depth, which is more than enough to visualise the methylation patterns of the entire bee brain. This approach offers a great potential to analyse the condition-specific brain methylation dynamics in the honey bee. These results are promising and suggest that technical issues are no longer a limiting factor in generating the methylation profiles of virtually all cell types in whole insect brains, or even selected regions of vertebrate brains. An important implication of our findings is that the epigenomic signatures in multicellular organs must be studied at the level of a specific cell group rather than using whole-brain extracts. A sufficient sequencing depth of long reads needs to be achieved to reveal all potential patterns.

## 4. Materials and Methods

### 4.1. Honey Bees and Brain Dissections

Honey bees were collected from our colonies located at the ANU Campus Field Station (Canberra, Australia). Newly emerged bees were labelled with paint and either transferred to small cages (15 per cage) or returned to the hive. We have used brains from young behaviourally naive bees and contrasted them with brains of mid-age bees and old bees involved in specific tasks, namely nursing (brood feeding) and pollen collection. The 7-day-old naive bees collected from the cages were not involved in any tasks and their brains were not exposed to external stimuli. The 93-day-old foragers carrying pollen were collected at hive entrance; 93-day-old “nurses” were collected from brood frames while feeding larvae (they had large hypo-pharyngeal glands and no visible wing damage in contrast to foragers that had withered glands and damaged wings). More details are provided in Table 2. Brains were dissected from freshly collected individuals chilled on ice. Special care was taken to prevent gland contamination, typically by removing the membrane surrounding the brain tissue. This step should not last longer than two minutes. Following the dissection, each brain was immediately frozen on dry ice until the extraction step. Five brains were pooled for each group before DNA extraction.

**Table 2:** Description of the honey bees used in this study.

Age of Bees (Days)	Functional Status	Comments
7	Caged bees reared in a dark incubator	No social colony-level environment, no specialised behavioural experience. Medium HPGs *
14	Hive bees that undertook orientation flights	These individuals were preparing for foraging. Medium HPGs
93	Mature hive nurse bees	Large HFGs, no indication of foraging experience (e.g., wing damage)
93	Mature foragers	Collected carrying pollen. Vestigial HPGs, evidence of wing damage
118	Very old nurse bees	Large HFGs, possibly some prior foraging experience, but external body damage, e.g., hair loss might be related to age

* HPG—hypopharyngeal gland. This gland is an indicator of a nurse or forager status [34]. A pool of five brains was analysed for each group.

### 4.2. DNA Bisulphite Conversion and Amplicon Preparation

A total of 1.5 µg of brain DNA [23] was bisulphite-converted using the QIAGEN Epitect^®^ Bisulfite Kit, as per the manufacturer’s protocol (Hilden, Germany). With this kit we typically perform two consecutive treatments to avoid incomplete conversion, especially in GC-rich regions [35]. The converted DNA was amplified via a nested PCR reaction (40 cycles) with gene specific primers (the bisulfite-sequencing primers have been described elsewhere [21]). The PCR products were purified utilising an Agencourt^®^ AMPure^®^ XP PCR Purification system (Beckman Coulter, Brea, CA, USA).

### 4.3. NGS Library Preparation and MiSeq Sequencing

Libraries were prepared from 500–600 ng of each amplicon utilising the NEBNext^®^ DNA Library Prep Master Mix for Illumina^®^, and NEBNext^®^ Multiplex Oligos for Illumina^®^ Index Primers Set 1 and Set2 (New England Biolabs, Ipswich, MA, USA). Size selection of the adaptor ligated DNA was performed using Agencourt AMPure XP beads (Beckman Coulter), with the bead:DNA ratio of the first bead selection 0.9×, followed by a second bead selection with a bead:DNA ratio at 0.2×. Each library was eluted in 30 µL of 0.1× TE, and the library size confirmed via agarose gel electrophoresis (we used Caliper LabChip GXII and the HT DNA High Sensitivity Assay (Midland, ON, Canada was performed on an Illumina MiSeq instrument using MiSeq Reagent Kit v3 (Illumina) and 600 cycles. A PhiX spike was added at 5% concentration as recommended by Illumina for low-diversity libraries.

### 4.4. Analysis of Bisulfite Sequencing Results

For each analysed sample, the frequency at which an mCpG occurred was calculated across all reads using custom Python scripts and open-source software. The process was comprised of two steps. In the first step, pairs of reads with the 30 nucleotide sequence starting at position 4 matching exactly the last 30 nucleotides of the primers used for the nested amplicon PCR were extracted from FASTQ files, aligned with an in silico bisulphite-converted genomic template using MUSCLE [35]; overlapping regions (if any) were proportionally truncated and, after removing all aligner-introduced gaps, both reads were combined into one continuous sequence and appended to a separate file (“extract file”) for each amplicon and each library/sample. In addition, a quality filter was applied, rejecting all sequences shorter than 90% of the length of the template or containing in excess of 5% gaps. In the second step, batches of sequences from the “extract” files were re-aligned with the template using MUSCLE (to eliminate any potential positional errors introduced by read indels), the aligned template sequence was used to calculate positional information of all the expected CpGs and SNPs and the positional data were used to score the methylation status (i.e., 0 for T and 1 for C occurring at a CpG position) for each combined read pair. The data were next appended to a separate table, and the unique methylation patterns were identified, and each unique pattern’s cumulative count was calculated for each amplicon and each library/sample. The pattern counts tables were next processed with the R package MPFE [36] to estimate the distribution of the methylation patterns, to eliminate spurious patterns and to generate the patterns plots. Several modifications to various steps of this protocol have been described in other papers [20,25,37].

## Supplementary Materials

The following are available online at https://www.mdpi.com/2075-4655/4/2/10/s1.
