# Supplementary material for: Exploring DNA Methylation Diversity in the Honey Bee Brain by Ultra-Deep Amplicon Sequencing
Source: Epigenomes. 2020 Jun 25;4(2):10. doi: 10.3390/epigenomes4020010 (PMC8594699; doi:10.3390/epigenomes4020010)
Supplement: Supplementary file 1 [file epigenomes-04-00010-s001.pdf]

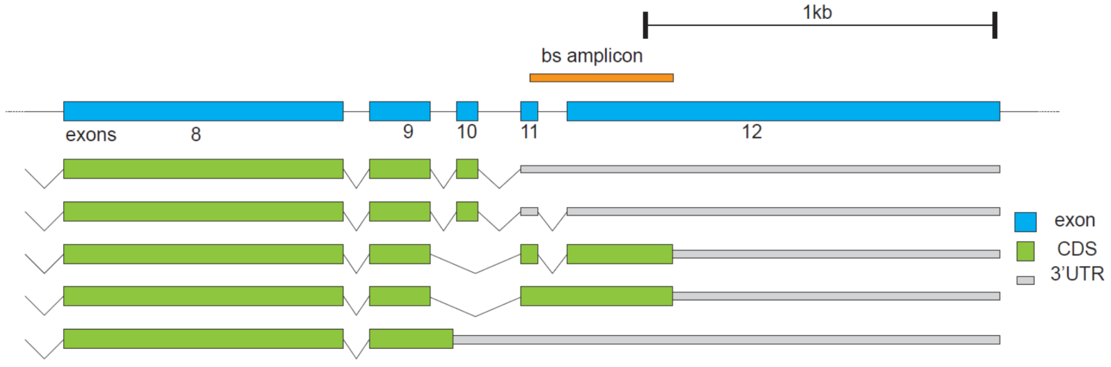

Figure S1. *Nadrin* gene model.

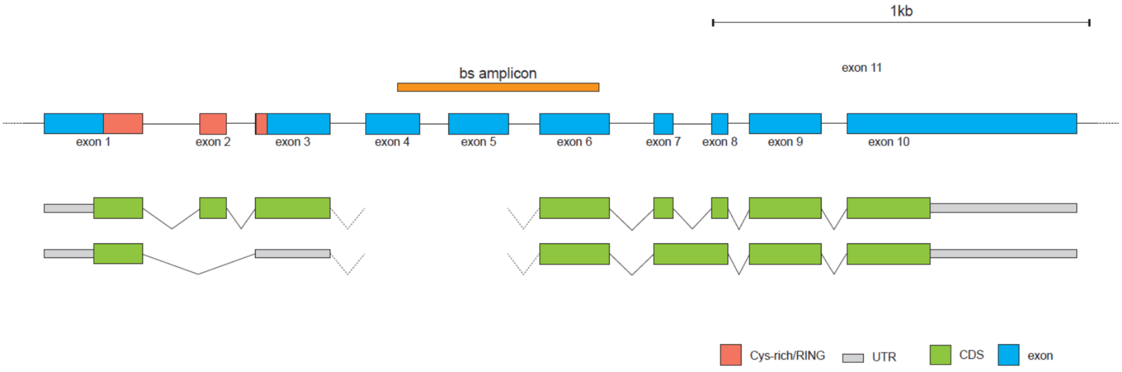

Figure S2. *Dynactin* gene model.

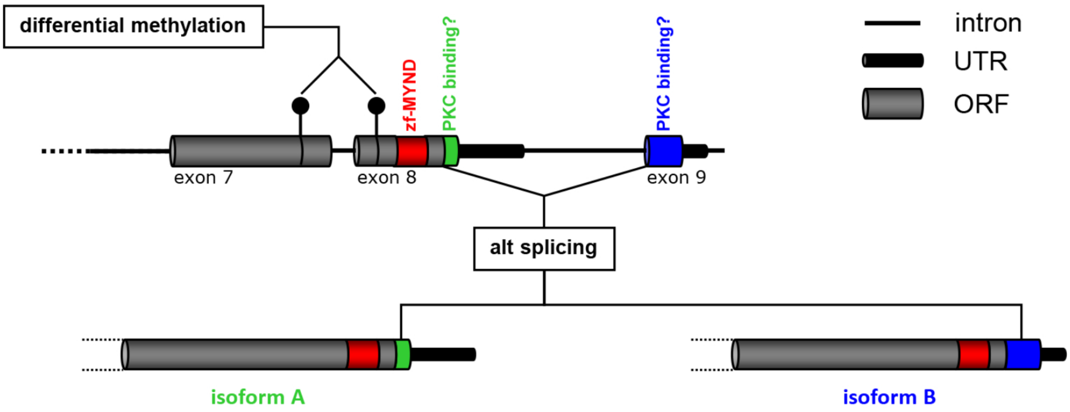

Figure S3. *pkcbp1* gene model showing two CpGs implicated in alternative splicing of exons 7, 8 and 9. Based on RNAseq and methylome data [16,21,24].

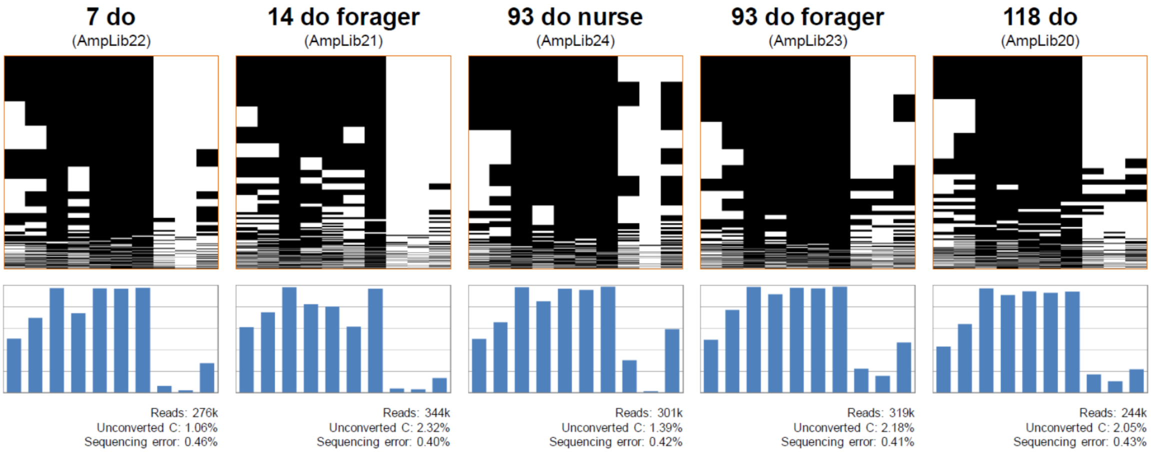

Figure S4. All methylation patterns for dynactin covered by Illumina MiSeq; "Frequency" denotes the pattern sorting. direction (i.e., most frequent patterns at the top). The lower panel shows the combined methylation level for each CpG. The age of bees used is shown above the top panel (see Table 2 for full description of the biological material). For details on dynactin gene structure and amplicon localisation see Figure S2 and Table 1. The number of reads per amplicon is indicted at the bottom. More details in caption to Figure 1.

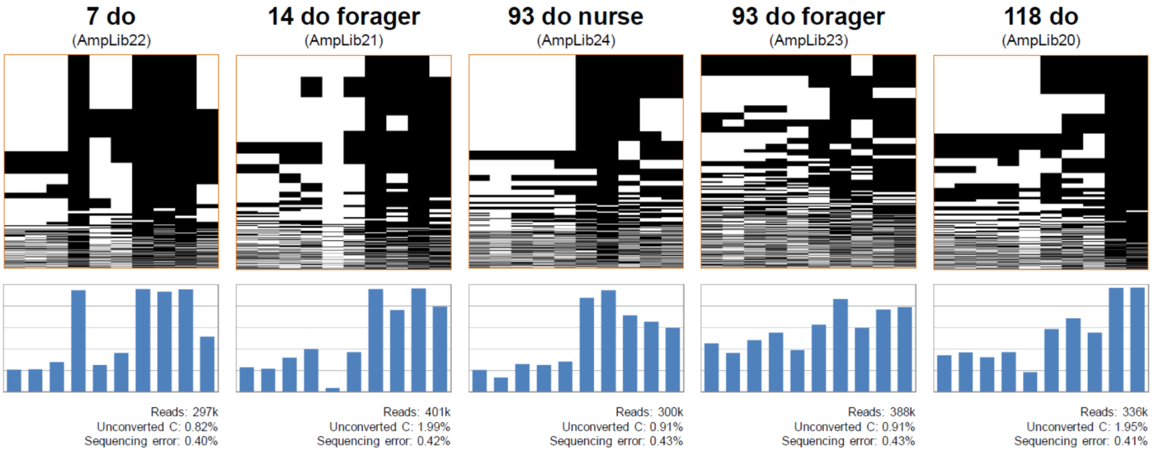

Figure S5. All methylation patterns for pkcbp1 covered by Illumina MiSeq; "Frequency" denotes the pattern sorting direction (i.e., most frequent patterns at the top). The lower panel shows the combined methylation level for each CpG. The age of bees used is shown above the top panel (see Table 2 for full description of the biological material). For details on dynactin gene structure and amplicon localisation see Figure S3 and Table 1. The number of reads per amplicon is indicted at the bottom. More details in caption to Figure 1.
